# Development of microfluidic devices for on-site water quality testing using glass molding process

**DOI:** 10.1007/s44211-023-00335-3

**Published:** 2023-04-27

**Authors:** Hidekatsu Tazawa, Tomomi Sato, Yu Sakuta, Ryo Miyake

**Affiliations:** 1 Institute of Microchemical Technology Co. Ltd., A-19 AIRBIC, 7-7 Shinkawasaki, Saiwai-ku, Kawasaki, Kanagawa 212-0032 Japan; 2 Department of Bioengineering, School of Engineering, The University of Tokyo, 7-7 Shinkawasaki, Saiwai-ku, Kawasaki, Kanagawa 212-0032 Japan

**Keywords:** Microfluidic devices, Water quality, Glass molding, Diamond-like carbon, Residual chlorine

## Abstract

The demand for multi-point water quality monitoring is increasing to solve the global problem of safe drinking water supply and environmental water contamination by industries. Therefore, compact devices are needed for on-site water quality analysis. On-site devices require low cost and high durability because they are placed outdoors, exposing them to strong ultraviolet rays and a wide range of temperatures. Our previous study reported on a compact and low-cost water quality meter that uses microfluidic devices with resin to monitor chemicals. In this study, we extended the fabrication range of the glass molding method to fabricate a glass microfluidic device with a 300 µm deep channel on a 50 mm in diameter substrate for constructing a low-cost and high-durability device. Finally, we developed a low-cost, highly robust glass device with a diamond-like carbon-coated channel surface to measure residual chlorine. The experimental results indicated that this device can endure outdoor conditions and be attached to small internet of things devices for analyzing chemical substances, such as residual chlorine.

**Graphical Abstract:**

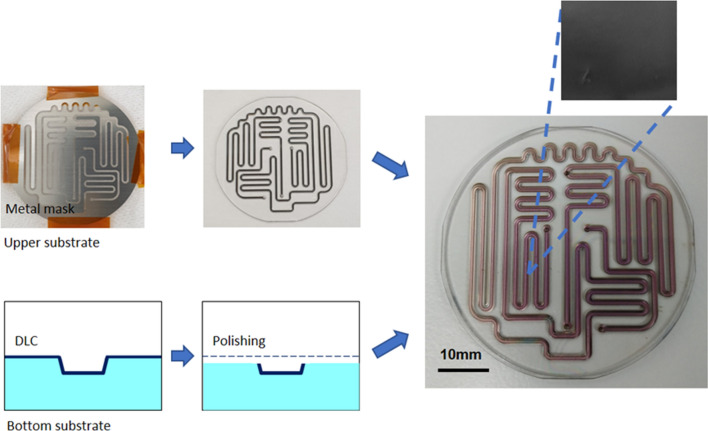

**Supplementary Information:**

The online version contains supplementary material available at 10.1007/s44211-023-00335-3.

## Introduction

Improving water quality is currently a global priority, including supplying safe drinking water and reducing environmental pollution caused by wastewater. Therefore, water analyzers are required to measure water quality on-site, such as at the water supply intake of each residence, agricultural water, and building water supply. Typically, the measurement points are located outdoors, which implies that the measurement devices should withstand the outdoor environment. Recently, compact and low-cost terminals with various sensors for water analysis have been developed [1–3]. These sensors measure flow rate, pH, turbidity, and dissolved oxygen (DO) levels [4], but not chemical levels. However, on-site multi-point monitoring of chemical substances is required. For instance, in the case of residual chlorine, water quality varies for every country or region. The concentration of residual chlorine in tap water is lower at the tap than that in the water pipe where it is measured. Therefore, the water pipe measurement does not reflect the actual concentration of drinking water. Measurements are preferably performed near the tap using a small, low-cost, Internet of Things (IoT) device [5].

Therefore, compact devices are required to measure the residual chlorine concentration at terminal water supply points and analyze large quantities of data. The use of electrode [6], biosensor [7], and *N*,*N*-diethyl-p-phenylenediamine (DPD) [8, 9] have been reported for small on-site measurement devices. However, the electrode method has developmental issues such as oxidation of the electrode [10], while biosensor methods are not robust because they depend on reaction principles [11]. Currently, the DPD method is the most widely used method for residual chloride measurement. However, its use of optical detection means the measurement device is required to have excellent optical properties.

In our prior research, we developed compact devices that enable the detection of chemical substances using resin-based microfluidic devices [12, 13]. Microfluidic devices enable chemical reactions and measurements in small flow paths of up to several hundred micrometers. They are suitable for on-site real-time measurements owing to their advantages, such as high-speed reactions, small device size, and small sample size. Moreover, water analysis is widely performed and the measurement devices are regularly exposed to intense outdoor UV light. They are also exposed to temperatures between − 20 and 80 °C locally. Additionally, contamination and mineral adsorption from polluted water must be prevented. Consequently, these devices are required to have high durability, antifouling properties, and flat surfaces. For this reason, the device’s material should be glass and resistant to UV radiation [14] and high temperatures [15]. However, the cost of manufacturing glass devices through wet etching is high owing to the considerable number of fabrication processes [16]. In addition, the wet etching method also creates small holes in the channels, called etch pits. The accumulation of contaminants in the etch pits [17] is a serious problem, particularly the formation of mineral scales on rough surfaces. To solve this, we previously developed a microfluidic device using the glass molding method (data not shown). With this method, channels are fabricated by pressing glass substrates at high temperatures and pressure. The mold shape is precisely pressed at the nanometer level in the original processing range. This method does not create pits and is mainly used to fabricate optical lenses [18, 19]. We optimized the channel design and processing conditions to use the glass molding method to fabricate microfluidic channels.

Consequently, the processing range was substantially expanded, making it possible to fabricate channels of several tens of micrometers on a 40 mm in diameter substrate. To measure residual chlorine concentration via the DPD method in a microfluidic channel, a long flow path is required for the reaction, which cannot be designed on a 40 mm in diameter substrate. In addition, a flow path with a depth of several hundred micrometers is also required to avoid sample blockage owing to contamination. Therefore, it is necessary to expand the processing size.

In this study, we examined and optimized the processing conditions of the glass molding process to fabricate flow paths with a depth of several hundred micrometers on a 50 mm in diameter substrate. Moreover, we developed a coating method that can help prevent the adsorption of minerals in the sample water and measured the residual chlorine in the sample water.

## Materials and methods

### Glass molding

**Fig. 1:**
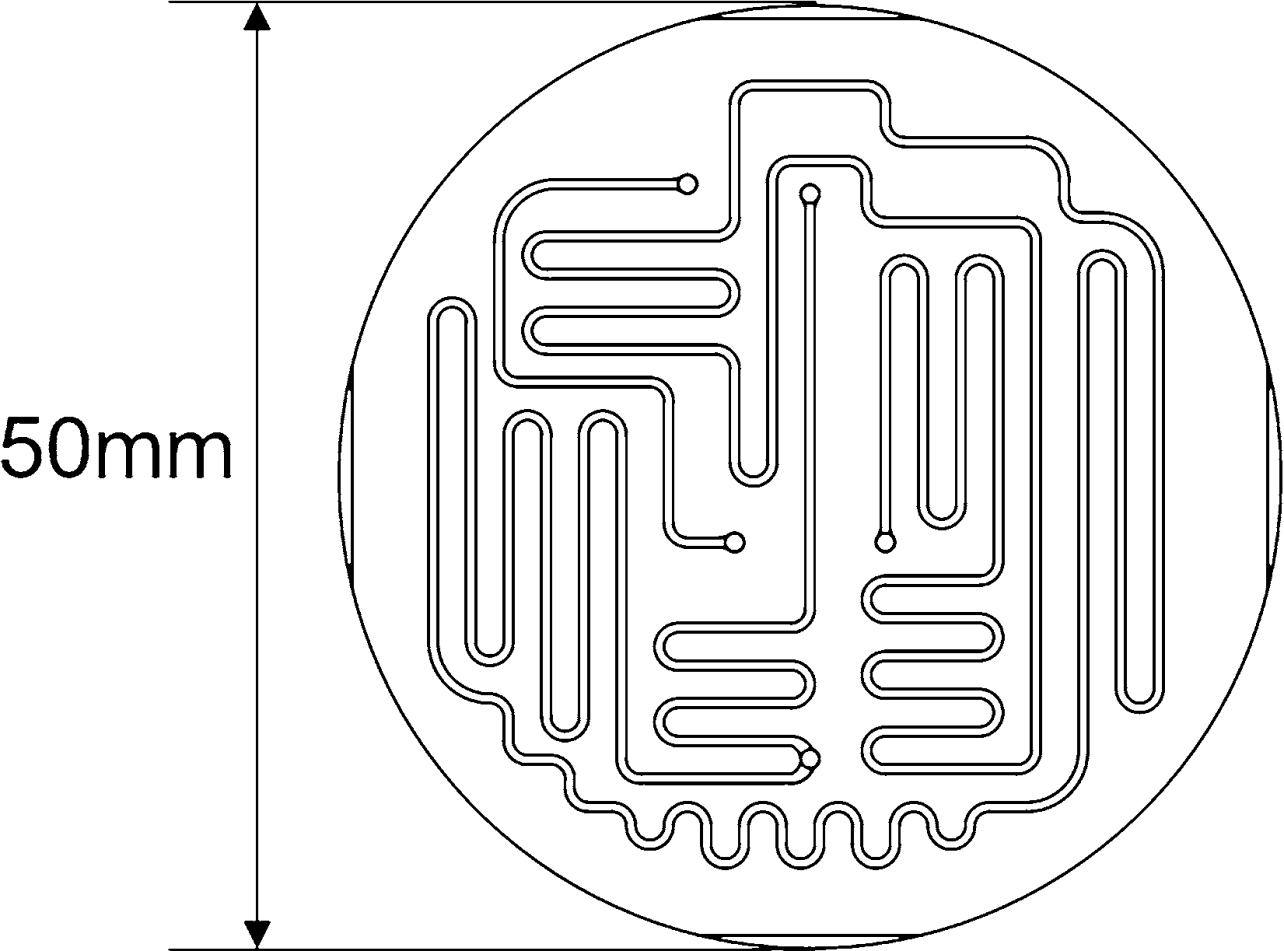
Design of molding device

The devices were fabricated through glass molding. The characteristics of each processing method are presented in Table 1. Milling [20–22] and wet etching [23, 24] were suitable for only a few prototypes; hence, the cost will not decrease even in mass production. Glass molding, which imparts a flat surface [25, 26], is suitable and inexpensive for mass production; however, it is difficult to fabricate deep channels. To address this problem, we developed a processing condition to fabricate deep channels through glass molding. We used K-PBK-40 (Sumita Optical Glass, Inc., Saitama, Japan) as the glass substrate for glass molding. The channel was designed to be 500 µm wide and 300 µm deep. The substrate pressing conditions were 560–600 °C and 1–3 kN. Moreover, to fabricate the long reaction channels, the substrate size was expanded to 50 mm in diameter and the channel design was employed throughout the substrate to balance the pressure during pressing (Fig. 1 design of the device).

**Table 1:** Characteristics of fabrication methods

Parameter	Milling [20–22]	Wet etching [10, 23, 24]	Glass molding [25, 26]
Fabrication range (μm depth)	100–300	1–250	1.2–
Defects	Generate	Generate	Not generate
Roughness (Ra, nm)	130–320	10–18	2–3

### Device coating

**Fig. 2:**
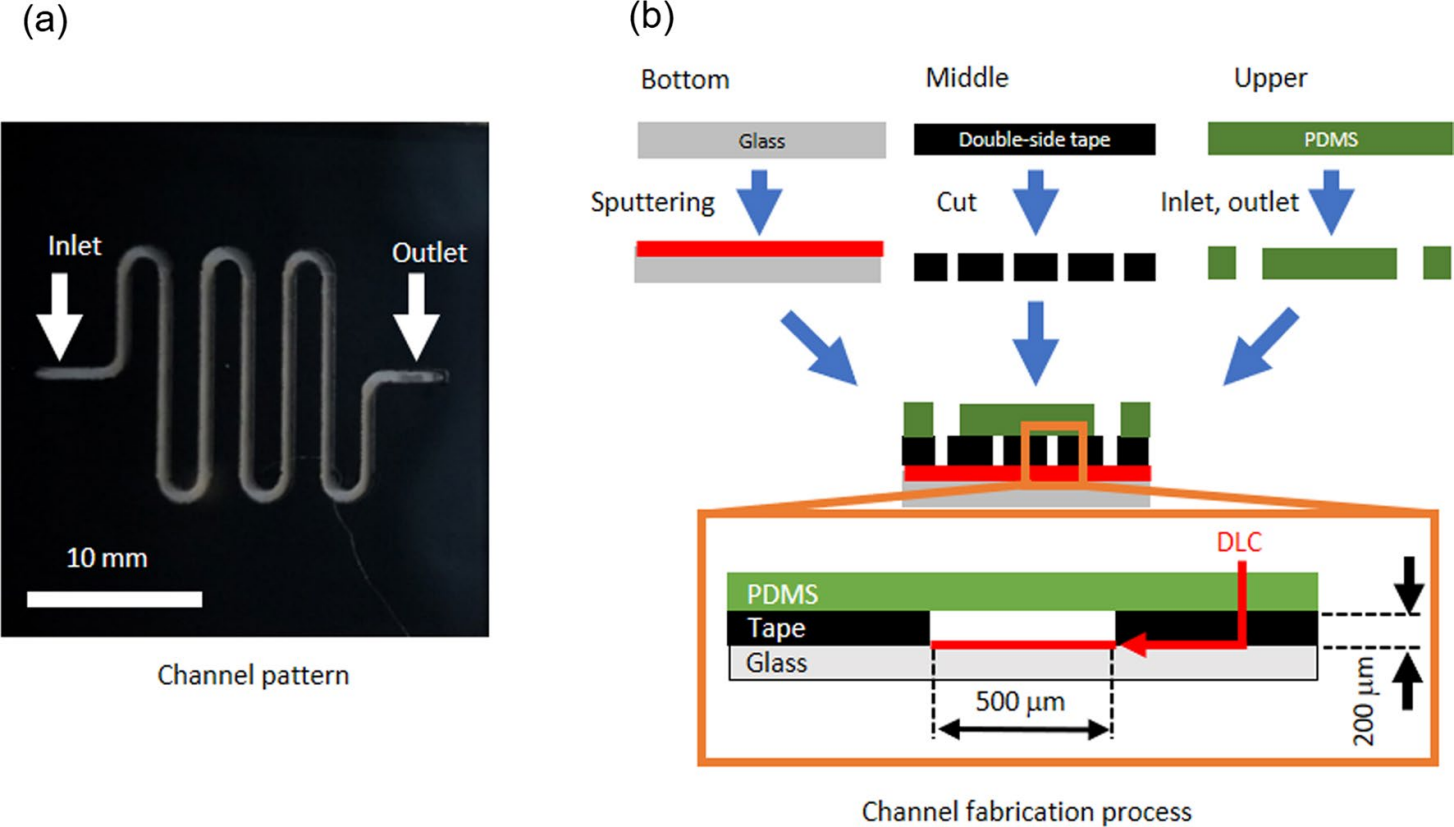
Construction of device to confirm coating effect. **a** Channel pattern and **b** channel fabrication process

**Fig. 3:**
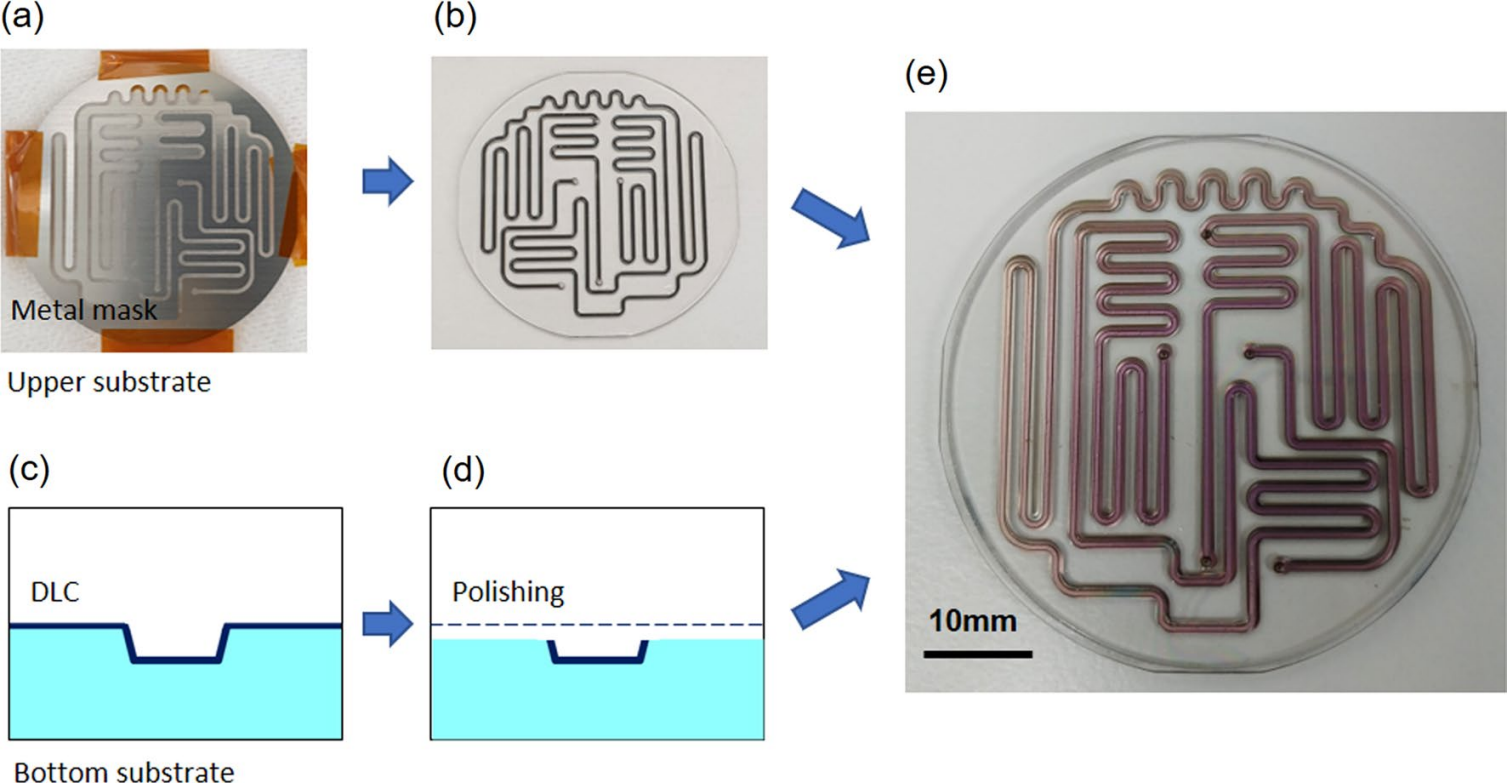
Schematic diagram of coating method for microchannel. **a** Metal mask for the upper substrate, **b** sputtered DLC using a metal mask, **c** cross-sectional view of a channel in the bottom substrate after DLC sputtering, **d** cross-sectional view of the channel after polishing, and **e** bonded device

**Fig. 4:**
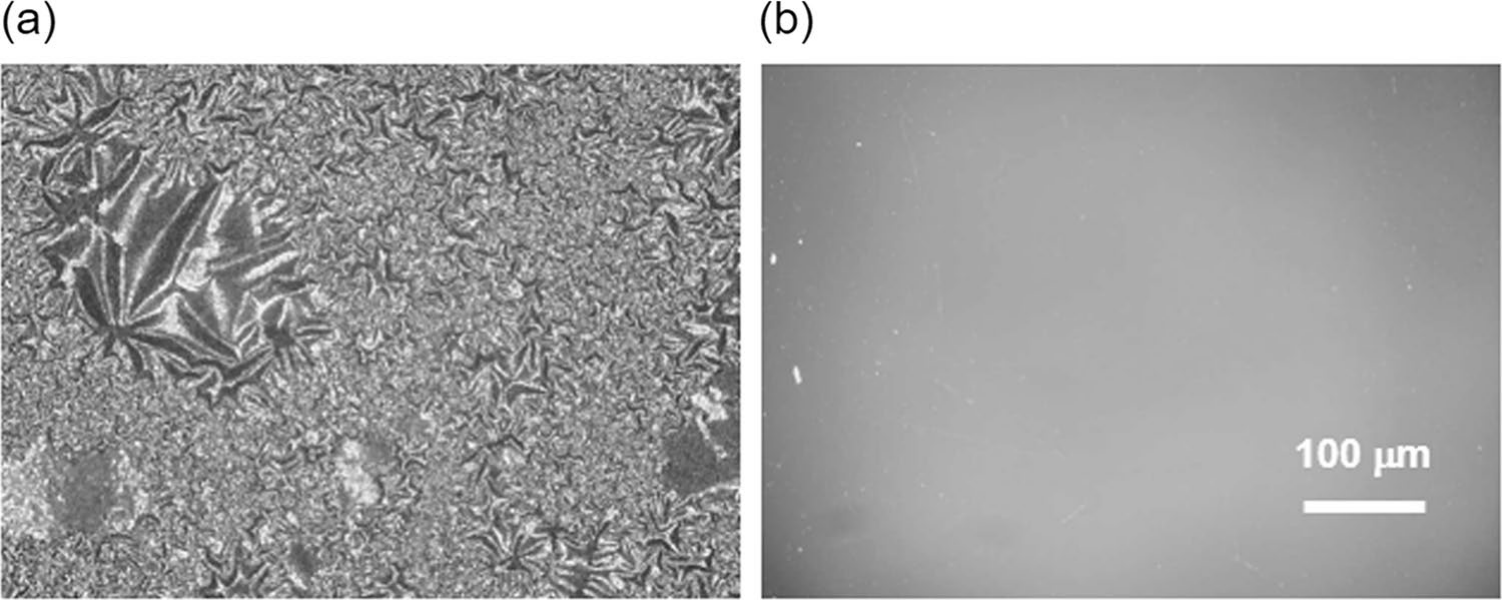
Comparison of base layer after heating. **a** Cr base and **b** Si base

Although glass is a low-adsorption material for many environmental contaminants [27], Ca and Mg are more easily adsorbed than plastics [28]. Therefore, the glass should be coated with low-frictional and low-adhesive material. Moreover, the coating material should be robust. Hence, we considered using diamond-like carbon (DLC) [29] to meet these requirements. To confirm the mineral adsorption inhibiting effect, an experimental was conducted using a DLC-coated glass substrate, as illustrated in Fig. 2. The evaluation device was constructed in a three-layer structure with top, middle, and bottom consisting of polydimethylsiloxane, double-sided tape, and glass, respectively. DLC was coated on the glass substrate by electron cyclotron resonance sputtering. Sputtering conditions were 2.5 kV, 3 × 10^–3^ Pa, 100 W, 1.5 mA/cm^2^, and the target-to-substrate distance was 95 mm. The layer thickness was 15 nm for the Cr substrate and 60 nm for the DLC. Microfluidic channels were created by hollowing out the double-sided tape with a laser cutter. For comparative purposes, DLC-coated and untreated substrates were prepared for the glass bottom plate substrate. Commercial mineral water (Contrex, Nestle Japan, Hyogo, Japan), which contains 468 mg/L of Ca and 74.5 mg/L of Mg, was used as the test sample. ImageJ (Rasband, W.S., ImageJ, U. S. National Institutes of Health, Bethesda, Maryland, USA, http://imagej.nih.gov/ij/, 1997–2012.) was used to measure the area of scales on the substrate. The schematic diagram and results of coating the channel with DLC are shown in Fig. 3. Figure 3a depicts the metal mask, fabricated from stainless steel, used for masking. The upper substrate was sputtered via the metal mask, as shown in Fig. 3b. The bottom substrate was also sputtered using the metal mask (Fig. 3c). Both substrates were coated via photolithography using a metal mask in a pattern 500 μm wider than the channels. The surface was then polished by a lap polishing process using cerium oxide to a depth of 50 µm, resulting in the channel formation, as shown in Fig. 3d. The upper and bottom substrates were aligned for bonding, as shown in Fig. 3e. An N_2_ substitution line was installed in the furnace for device bonding, and bonding was conducted within an N_2_ environment. The underlying metals, Cr and Si, were heated at 550 °C and compared, as shown in Fig. 4. The DLC surface on the Cr layer exhibits roughening after heating, as shown in Fig. 4a, in contrast to the smooth DLC surface on the Si layer, as shown in Fig. 4b. Hence, Si was selected as the underlayer for the substrate. In addition, complete bonding was not possible when the substrates coated with DLC on all surfaces were bonded together, as shown in Online Resource 1. Therefore, the device bonding was conducted using the DLC-coated substrates patterned with masking.

### Holder for microfluidic device

**Fig. 5:**
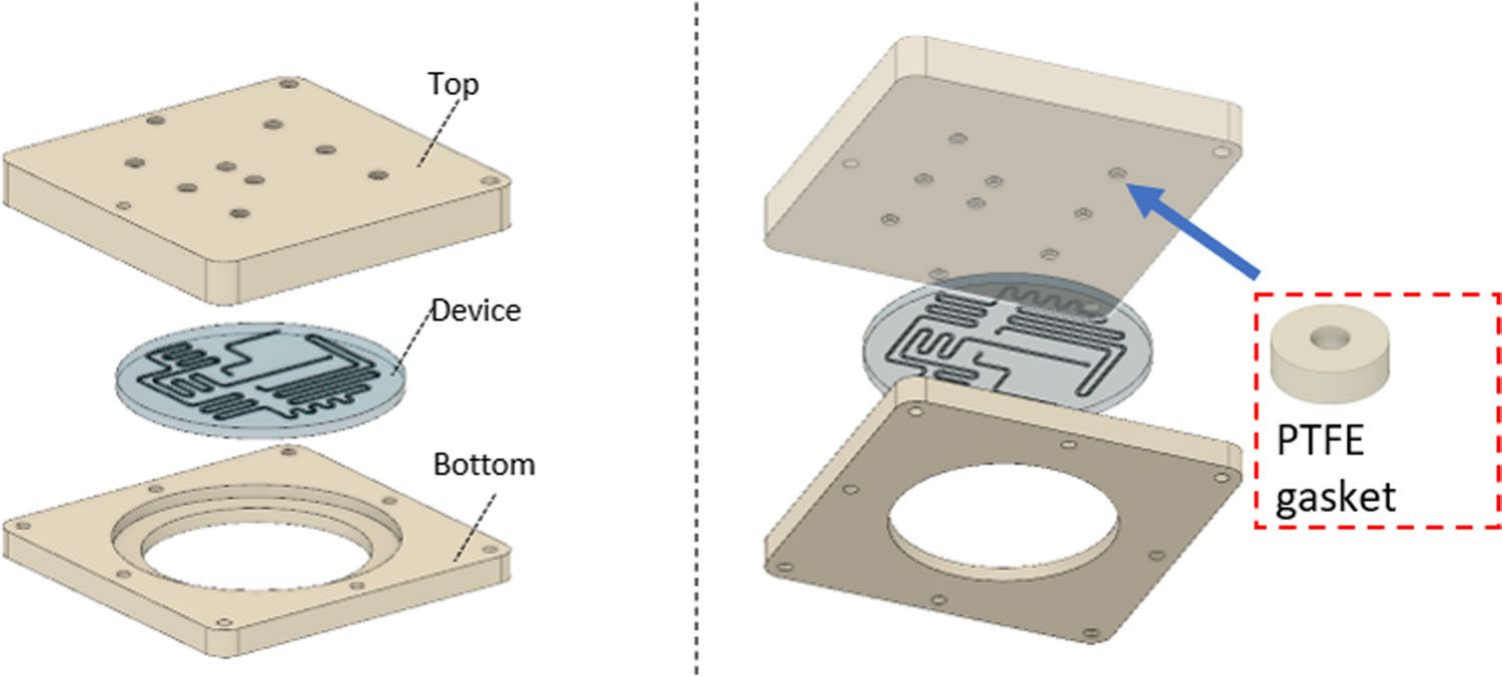
Design of device holder

The holder was fabricated by machining. Polyphenylene sulfide (PPS) was chosen as the injection molding material for mass production. A polytetrafluoroethylene (PTFE) gasket was used to connect the holder to the device so that tubing could be attached without disassembling after the device was set, as shown in Fig. 5. The pressure resistance was then evaluated.

### Water flow experiment

To evaluate the tubing and channels for pressure resistance and blockage, a flow-through experiment was conducted on the bonded device. We determined whether there was leakage and if precipitates were adsorbed in the flow channel from tap water flowing at 40 mm/min for one week. Leakage was confirmed by pumping and visually inspecting the device after the experiment was completed. Next, the water flow experiment was conducted using Contrex at 15 µL/min for 10 days to evaluate mineral adsorption. Moreover, a chlorine standard solution was passed through the device, and the chlorine content was measured using the Digital Pack Test (Kyoritsu Chemical-Check Lab. Corp., Tokyo, Japan). Residual chlorine standard samples were prepared with sodium hypochlorite solution (OYALOX Co., Ltd., Tokyo, Japan). The liquid was pumped at a flow rate of 15 µL/min by water head difference and measured with a spectrophotometer (DPM2-ClO-DP) after collecting 1.5 mL.

## Results

### Device fabrication

#### Device fabrication by glass molding

**Fig. 6:**
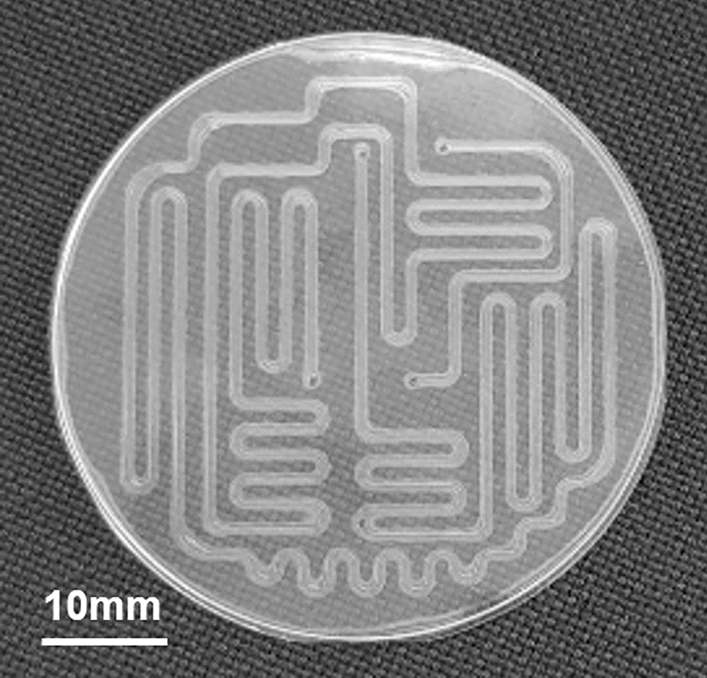
Device fabricated using glass molding method

**Fig. 7:**
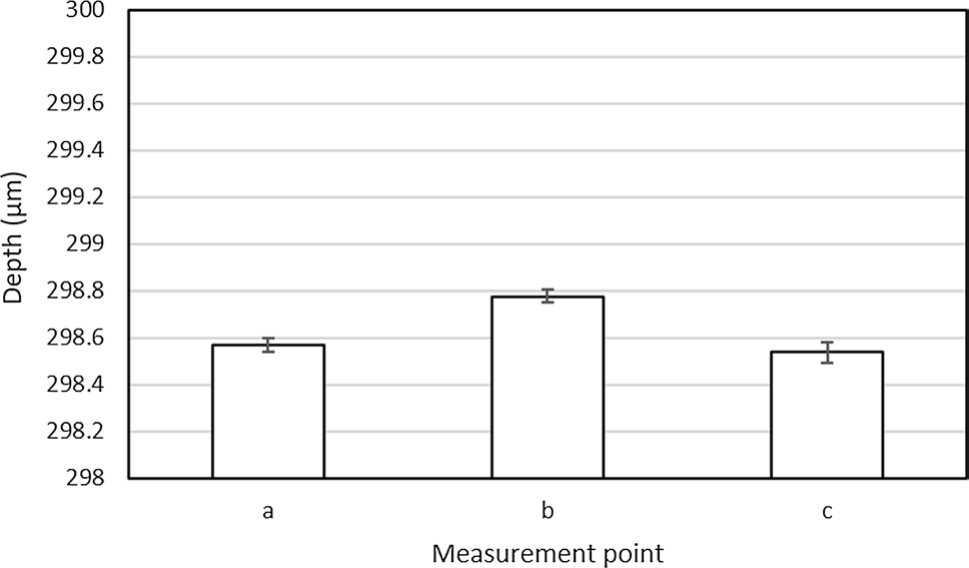
Precision of channel depth. Data represent the mean ± S.E. (*n* = 6)

As the mold is often broken when the glass substrate is extracted, we considered conditions that would create a channel with minimal glass deformation to prevent mold destruction by glass adsorption. The results of the pressing conditions of the glass substrates are shown in Table 2. The glass substrate started producing channels at 580 °C, and bubbles started to form at 600 °C. The device cracked at temperatures lower or higher than the optimum point, as shown in Online Resource 2. Next, pressure conditions were examined at 580 °C in the press. According to the results presented in Table 2, the channels could be fabricated at 3.0 and 5.0 kN without bubbles. Therefore, the press conditions for device fabrication were selected as 580 °C and 3.0 kN. In addition, the bonding conditions with the blank substrate were set to 560 °C and 0.98 kN. The device fabricated under these conditions could be fluidized at 0.8 MPa without damage. As shown in Fig. 6, we fabricated devices with a substrate size of 50 mm in diameter and a channel depth of 300 µm, and as shown in Fig. 7, the channel depth precision was less than 1 µm. In this device, the mold release conditions were optimized to make it easier to release the substrate from the mold. As a result, we could process a substrate diameter of 40 mm and a channel depth of 100 µm or more, which had previously been the largest processing sizes available. Therefore, even with this substrate size and channel depth, the channels could be pressed without damaging the substrate and deep flow paths could be fabricated on large-diameter substrates.

**Table 2:** Molding process parameters

Temperature (℃)	Molding	Bubble
560	☓	○
570	☓	○
580	○	○
590	○	○
600	○	☓

Pressure (kN)	Molding	Bubble
1.0	☓	○
3.0	○	○
5.0	○	○

#### Pressure resistance of holder

The results of the liquid flow test and pressure resistance measurements are shown in Table 3. The results show that a gasket with a thickness of 1.2 mm in a 1.0 mm inlet depth did not leak even at a pressure of 5 bar. Therefore, we used a 1.2 mm thick gasket in this study.

**Table 3:** Gasket thickness for sealing between the device and holder

Thickness (mm)	0.1 bar	1 bar	5 bar
1.0	☓		
1.1	○	○	☓
1.2	○	○	○

#### DLC coating effect

**Fig. 8:**
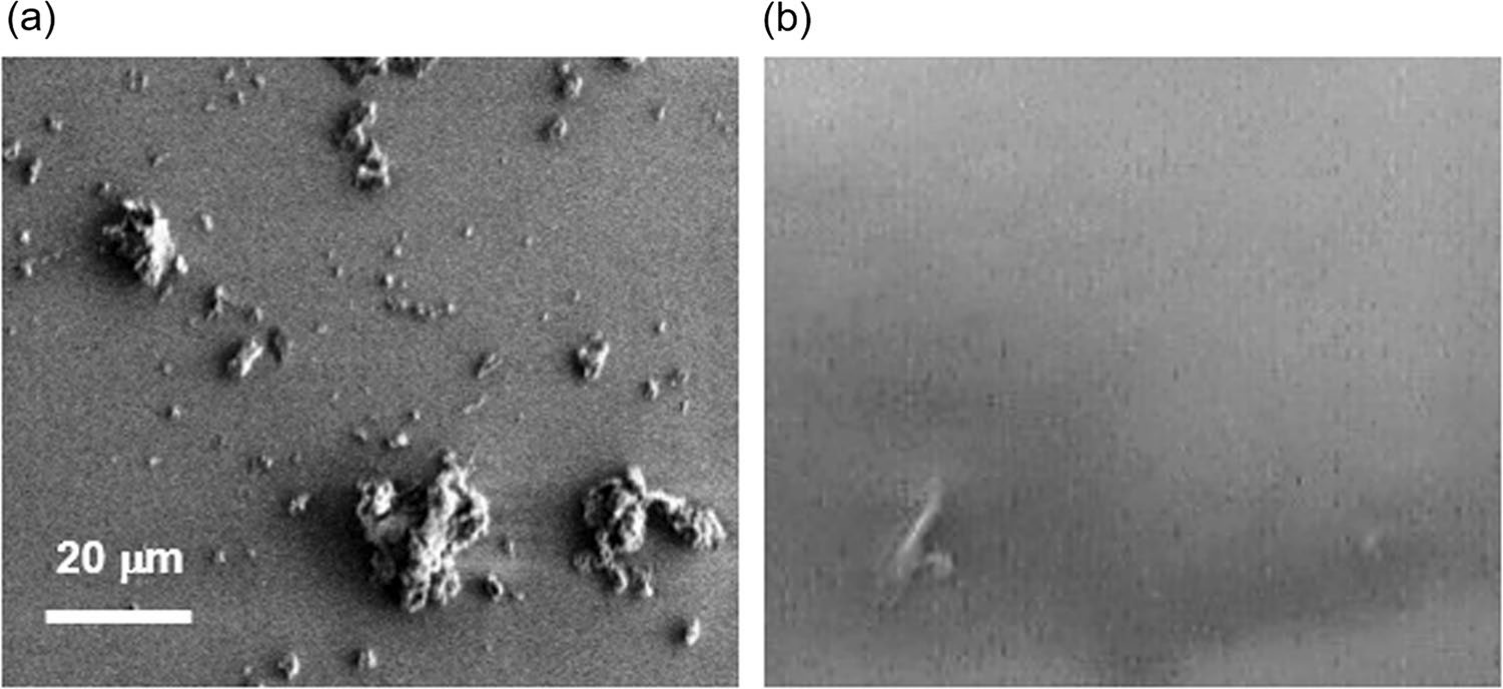
SEM images for comparison of coating efficiency. **a** Non-coated substrate and **b** DLC-coated substrate

**Fig. 9:**
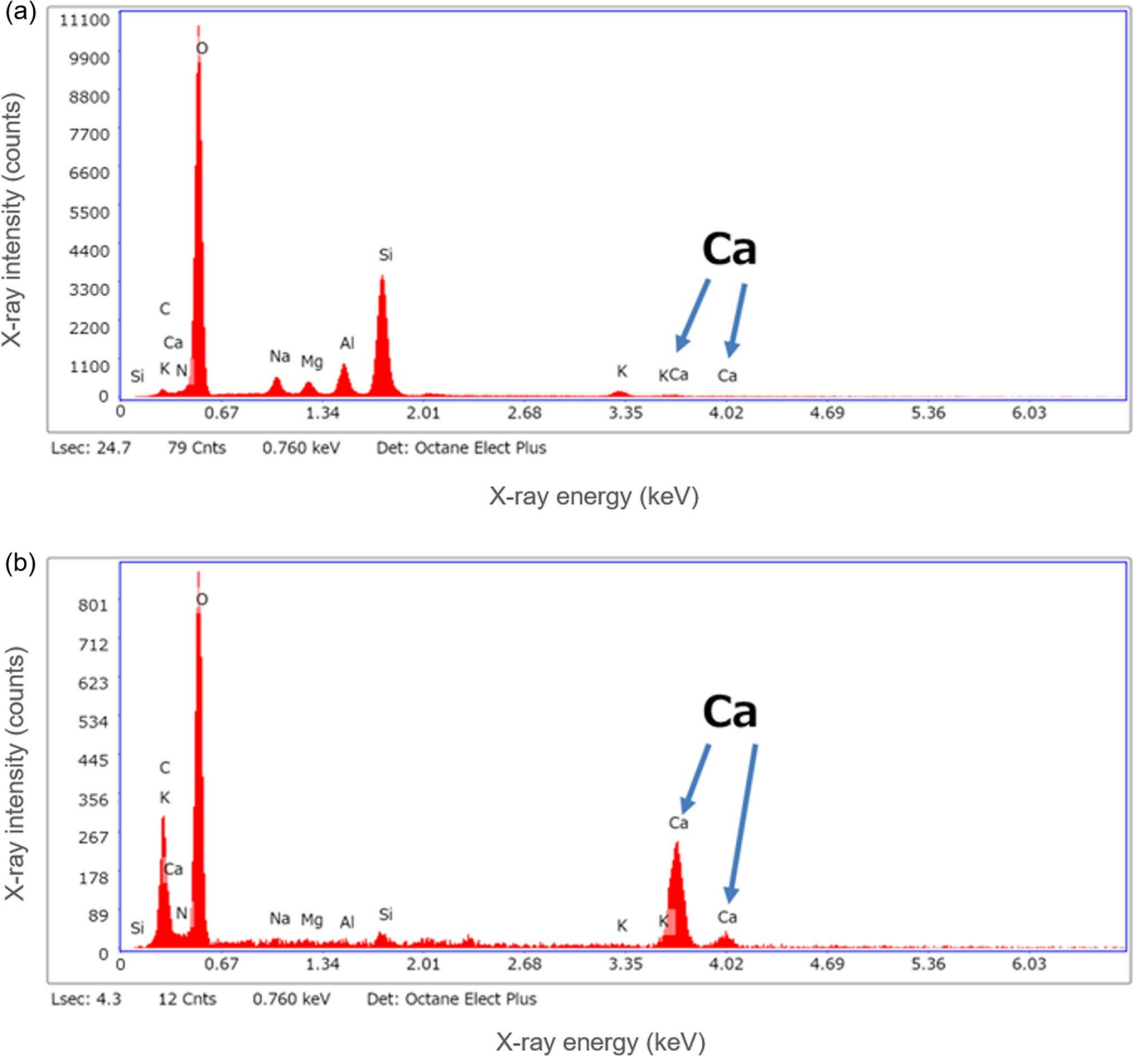
Comparison of channel surface using energy dispersive X-ray spectroscope. **a** Bare substrate and **b** scale areas

**Fig. 10:**
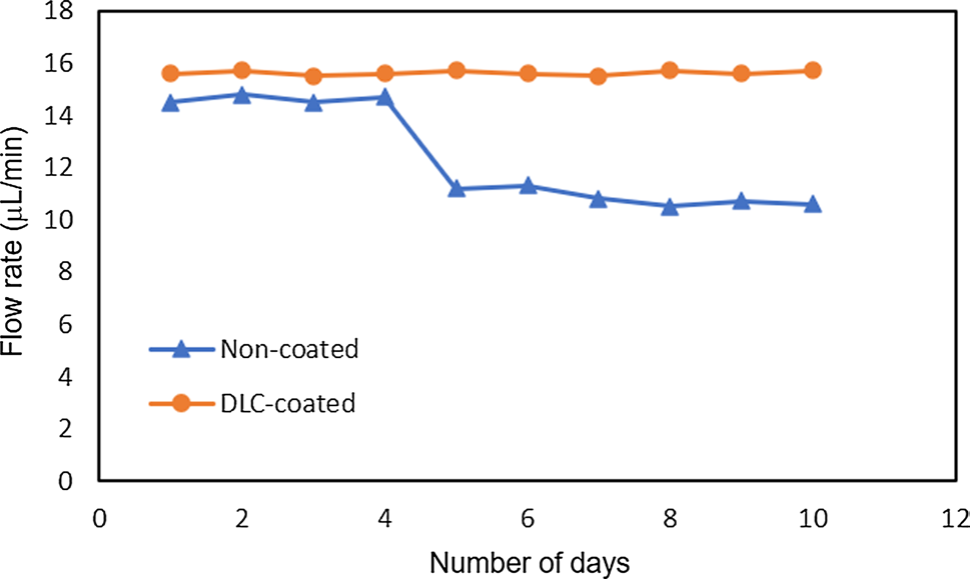
Flow rate as a function of the adsorption efficiency

The results of one week of water flow with high-hardness water are shown in Fig. 8. Significant scale adhesion was observed on the uncoated substrate, presumably because of the presence of minerals. In contrast, the number of adsorptions was less on the DLC-coated substrate. The precipitated material was analyzed using an energy-dispersive X-ray spectroscope. Measurements were compared between the precipitation-only and precipitation-free areas, as shown in Fig. 9. The measured precipitates contained more K and Ca than the precipitation-free area indicating that precipitates containing K and Ca are easily adsorbed on substrates without DLC coating. Image measurement results showed that the precipitated area was 1.15% for the DLC-coated substrate and 11.89% for the uncoated substrate in a 100 μm^2^ measurement area. The flow tests using tap water were conducted at 40 mm/min and the water flowed through the channel without leaking or peeling the DLC. Furthermore, no scale adhesion was observed via an optical microscope after one week. The flow test results using Contrex are shown in Fig. 10. The flow rate of the non-coated devices decreased by approximately 20% from day five. In contrast, the flow rate of DLC-coated devices did not decline even after 10 days. This can be attributed to the mineral scale that had peeled off from the flow channel and clogged it. The scale was been observed for 24 h in the bulk test, and the flow stripped it off due to the channel. The channels are micro-sized, which means that small amounts of mineral scales can easily block the channels. Therefore, DLC coating was effective in preventing the adhesion of mineral scale.

### Residual chlorine measurement

**Fig. 11:**
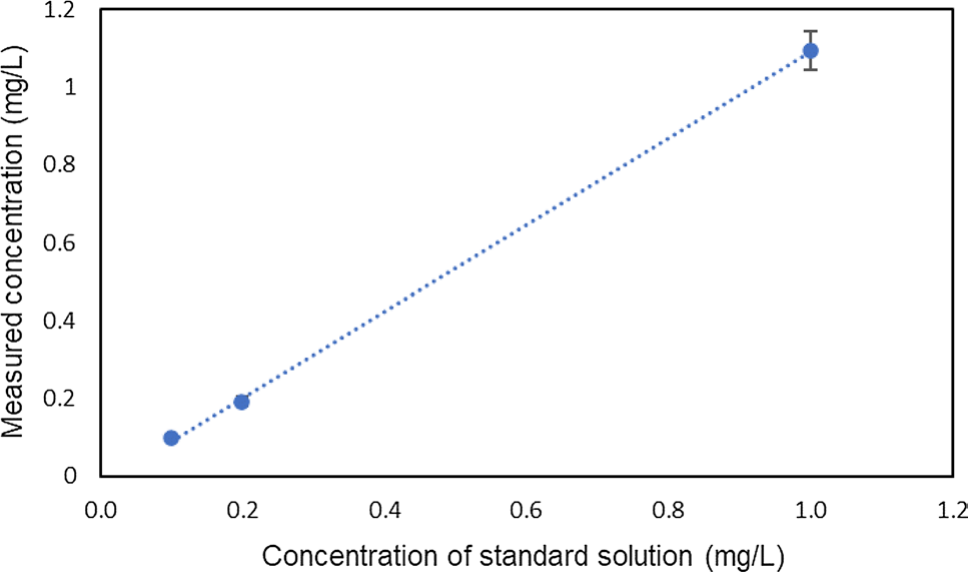
Chlorine standard solution analysis

The DPD method is inhibited by metals and calcium ions; therefore, the residual chlorine of a standard concentration was measured using the device to confirm the effects of Si used in the device and the Ca scale adsorbed to the channel. The results of the standard chlorine solution measured through the DLC-coated device are shown in Fig. 11. For 0.1, 0.2, and 1.0 ppm residual chlorine measurements, all the DLC-coated devices demonstrated similar values. Therefore, they can be used for measurements in the range of approximately 0.1–1 ppm using the DPD method.

## Discussion

### Glass molding device

Glass is the most suitable material for microfluidic devices used for water analysis because of its durability and antifouling properties. However, it is difficult to fabricate. Currently, milling and wet etching are the main processing methods used in glass fabrication. However, milling is processed by grinding the glass surface using a tool, which results in a rough surface [22], and the masking of the substrate used for wet etching begins to peel off at a depth of approximately 250 µm or deeper. Additionally, it is impossible to prevent the formation of pits on the channel surface during etching [10]. Moreover, these methods are expensive and equivalent low-cost manufacturing methods have not yet been established. In contrast, glass molding does not cost much and produces a flat surface; however, creating large-diameter substrates and deep channels is difficult. Consequently, the substrate and channel dimensions were limited to 40 mm in diameter, 100 μm in width, and 100 μm in depth, as in our previous study [data not shown]. To fabricate deep channels on a large substrate, we dispersed the channels across the entire substrate to reduce the bias of the pressing pressure. Thereafter, the press temperature of the fabrication process was reduced to the maximum extent.

### Holder

The holder should be made of a material that does not change its shape at temperatures between − 20 °C and 80 °C. PPS is a resin that does not change its shape significantly with temperature, and its linear expansion coefficient (*α*) is 2.5 × 10^–5^/ °C. By contrast, PTFE has a very high *α*, ranging from 12.4 to 79 × 10^–5^/ °C, depending on the temperature. The actual dimensional (*L*) change of the gasket has been calculated, and the dimensional difference (Δ*L*) owing to temperature difference (Δ*T*) is expressed as

1 $$\Delta L=\alpha L\times\Delta T$$

Using Eq. (1), the Δ*L* values for − 20 °C and 80 °C are 7.3 µm and 8.2 µm, respectively. This indicates that the thickness of the 1.2 mm gasket is less affected by shape change with temperature and can maintain sufficient sealing and pressure resistance.

### DLC coating and substrate bonding

Comparing Cr and Si as the base layer of DLC revealed that the Cr base substrate became wrinkled after heating under vacuum and N_2_ conditions. DLC typically requires an intermediate layer owing to the high residual stress within the film and lack of adhesion to the substrate. In general, Cr is used for ferrous substrates; however, Si was more suitable in this case. This can be explained by the difference in the thermal expansion coefficients of Cr and Si. The *α* (× 10^–6^/°C) of Pyrex glass, Si, and DLC are similar at 3.2, 2.4, and 2.0, respectively, whereas the coefficient of Cr is 4.9. Consequently, it is believed that wrinkles form on the DLC surface because of the difference in the expansion coefficient. Based on these findings, Si was chosen as the base layer. The DLC material is carbon, which is oxidized and vaporized by oxygen in atmospheric conditions above 550 °C. Devices are bonded by sandwiching the substrate between flat plates and applying pressure and heating. In this process, the plate closes the introduction port of the channel. Even if bonding is performed in a vacuum furnace, it is difficult to completely remove air from the channel during bonding, causing the DLC to become oxidized and disappear. As a countermeasure, the furnace was filled with N_2_ before bonding and even though the channel inlet was closed, bonding was possible without oxygen.

### Residual chlorine measurement

The actual concentration of residual chlorine in tap water cannot be determined unless it is measured at each tap. Therefore, there is a need to develop a device that can be installed near the tap. The proposed device is suitable and effective in this regard. Moreover, if this device is used as an IoT device, it is possible to map the concentration of each area, also making it suitable for big data analysis. In addition, it has the advantage of being dirt resistant, making it possible to install it for long periods in otherwise difficult-to-install or replace locations, such as deep in the mountains. The needs of these locations are not limited to residual chlorine. It is also expected to be suitable for the measurement of other chemicals.

## Conclusion

In this study, we developed a low-cost glass microfluidic device by expanding the fabrication range of the glass molding process. The device was fabricated with 200 µm deep channels on a 50 mm in diameter substrate, and the channels were coated with DLC and bonded together. In addition, the holder for the tubing with a pressure resistance of up to 5 bar was also developed, and flow experiments were conducted. As a result, the flow rate was unchanged after flow testing water samples containing high mineral content for 10 days. Furthermore, we confirmed that the developed device could be used to measure residual chlorine in the range of 0.1–1 ppm. In the future, we plan to add an IoT communication function to this device and install it outdoors to measure residual chlorine. Moreover, the measurement of other chemical substances will also be considered.

## Supplementary Information

Below is the link to the electronic supplementary material.

Supplementary file1 (PDF 221 KB)

## Data Availability

The source data for figures are available on request from the corresponding author R, Miyake.
