# Supplementary material for: Development of microfluidic devices for on-site water quality testing using glass molding process
Source: Anal Sci. 2023 Apr 27;39(8):1269–77. doi: 10.1007/s44211-023-00335-3 (PMC10359213; doi:10.1007/s44211-023-00335-3)
Supplement: Supplementary file 1 — Supplementary file1 (PDF 221 KB) [file 44211_2023_335_MOESM1_ESM.pdf]

Supplementary Information

Development of microfluidic devices for on-site water quality testing using glass molding process

Analytical Sciences

Hidekatsu Tazawa*, Tomomi Sato**, Yu Sakuta* and Ryo Miyake[†]**

**Institute of Microchemical Technology Co. Ltd., A-19 AIRBIC, 7-7 Shinkawasaki, Saiwai-ku, Kawasaki, Kanagawa 212-0032, Japan*
***Department of Bioengineering, School of Engineering, The University of Tokyo, 7-7 Shinkawasaki, Saiwai-ku, Kawasaki, Kanagawa 212-0032, Japan*

[†]Corresponding author
E-mail: trmiyake@mail.ecc.u-tokyo.ac.jp

Device with all surface coatings was impossible to bond.

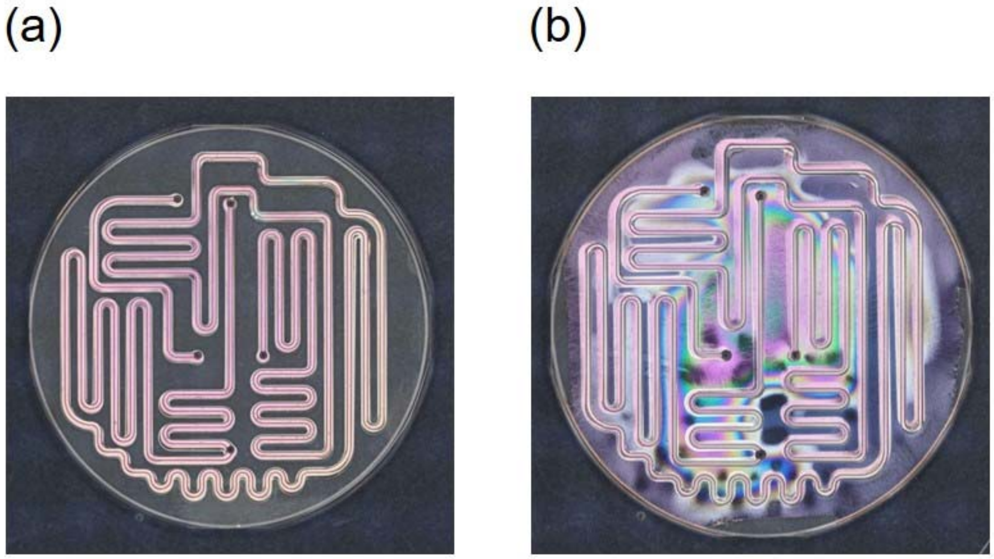

Fig. S1 Comparison of DLC coating method and bonding. (a) Coated substrates patterned with masking and (b) All surfaces coated substrate

The device cracked at temperatures lower or higher than the optimum point.

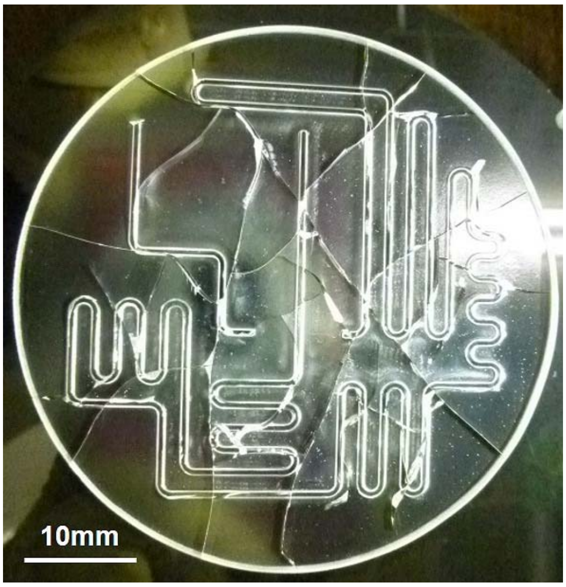

Fig. S2 Substrate with failed molding process.
